# Current Perspectives on GLP-1 Agonists in Contemporary Clinical Practice from Science and Mechanistic Foundations To Optimal Translation

**DOI:** 10.1007/s11883-025-01350-7

**Published:** 2025-10-09

**Authors:** Husam Abu-Nejim, Richard C. Becker

**Affiliations:** 1 The Warren Alpert Medical School of Brown University, Providence, RI USA; 2 University of Cincinnati College of Medicine, 231 Albert Sabin Way, Cincinnati, OH 45267 USA

**Keywords:** Atherosclerotic cardiovascular disease, Microvascular disease, Diabetes mellitus, Thrombosis, Chronic kidney disease, GLP-1R agonists

## Abstract

**Purpose of Review:**

This review provides a comprehensive and scholarly examination of glucagon-like peptide-1 receptor (GLP-1R) agonists, tracing their evolution from glycemic control agents in diabetes mellitus (DM) to multifaceted therapeutics with expanding indications in cardiovascular, renal, and metabolic health. We explore the underlying biological mechanisms, summarize clinical trial evidence, and highlight emerging applications in non-diabetic populations. Recent developments underscore the relevance of GLP-1R agonists in addressing the complex interplay of cardiovascular-kidney-metabolic (CKM) syndrome, microvascular dysfunction, and metabolic-associated steatohepatitis (MASH). We also discuss combination therapies and strategies to mitigate muscle mass loss during treatment and calls for targeted research, improved clinical education, and policy reforms to optimize the translational potential of GLP-1R agonists in both individualized care and population health.

**Recent Findings:**

Diabetes mellitus currently affects over 422 million individuals worldwide, with projections indicating a rise to 783 million by 2045, representing 10.5% of the global adult population. Common comorbidities include chronic kidney disease (CKD) and atherosclerotic cardiovascular disease (ASCVD), which collectively impact nearly one-third of individuals with DM. The growing prevalence of metabolic disease, CKD, and ASCVD have prompted investigation into the role of GLP-1R agonists in mitigating cardiovascular and metabolic risks, particularly within the framework of Cardiovascular-Kidney-Metabolic (CKM) Syndrome, irrespective of diabetic status. Emerging evidence, foundational science, and transformative knowledge of mechanisms of action further support the expansion of therapeutic indications for this drug class.

**Summary:**

Although GLP-1R agonists were originally developed for glycemic control in DM, their mechanistic versatility has enabled broader application across a spectrum of cardiovascular, cerebrovascular and metabolic disorders. This review traces the trajectory of their development and highlights opportunities for more expansive translational use in both clinical and population health settings. We also address current barriers to implementation and evidence-based use, ongoing clinical trials, and future directions, including combination therapies that may enhance efficacy and patient outcomes.

## Introduction

Diabetes mellitus (DM) affects over 422 million individuals globally, with projections indicating a rise to 783 million by 2045, representing 10.5% of the adult population. The burden of DM is compounded by common comorbidities such as atherosclerotic cardiovascular disease (ASCVD) and chronic kidney disease (CKD), which affect nearly one-third of individuals with DM [1, 2].

The increasing prevalence of DM, ASCVD, and CKD necessitates therapeutic strategies that are not only effective and safe but also accessible and scalable. GLP-1 receptor (GLP-1R) agonists, originally developed for glycemic control in patients with type 2 DM (T2DM), have demonstrated broad mechanistic versatility, enabling their application across a spectrum of cardiometabolic disorders. Their benefits extend beyond glucose regulation to include weight loss, blood pressure reduction, anti-inflammatory effects, and cardiovascular protection.

The primary objective of our focused review is to illuminate the scientific underpinnings and translational potential of GLP-1R agonists as a foundation for advancing clinical practice and achieving high-level patient care. By bridging rigorous mechanistic insights with real-world therapeutic applications, we aim to underscore how a deep understanding of GLP-1 biology—spanning metabolic, cardiovascular, inflammatory, and neurohormonal pathways—can inform more precise, effective, and individualized treatment strategies. Optimal translatability is not merely a matter of applying evidence; it involves aligning pharmacologic properties, patient phenotypes, and evolving clinical needs to maximize therapeutic benefit. In doing so, we advocate for a paradigm that moves beyond traditional indications, embracing the full spectrum of GLP-1R agonists in areas such as primary prevention, microvascular protection, and metabolic liver disease, and metabolic disorders that include cardio-kidney-metabolic (CKM) syndrome—a systemic disorder characterized by interrelated metabolic, renal, and cardiovascular dysfunction and a broader cardio-renal-metabolic axis that includes central obesity, insulin resistance, dyslipidemia, hypertension, and pre-diabetic states. This approach not only enhances outcomes but also sets a new standard for evidence-based, mechanism-driven care.

Last, we call upon clinicians, researchers, regulators, and industry stakeholders to address existing gaps in accessibility and explore opportunities for enhancing the use of GLP-1R agonists in both individualized care and broader public health contexts.

## Milestones in Glucose Homeostasis: A Foundation for GLP-1R Agonist Development

Studies have shown that oral glucose intake elicits a more robust insulin secretion response compared to intravenous glucose delivery, despite identical glucose concentrations [3]. This phenomenon, referred to as the “incretin effect,” was subsequently attributed to gastrointestinal and endocrine factors. The first factor identified was gastric inhibitory polypeptide (GIP), which was thought to increase glucose-stimulated insulin secretion. However, it was found that the incretin effect remained preserved even after the removal of GIP in experiments. Glucagon-like peptide (GLP) type 1 was subsequently identified as another active incretin [4].

In the 1980 s, researchers identified GLP-1 in the gut and discovered its role in enhancing insulin release while simultaneously suppressing glucagon secretion [5]. In 2005, the first GLP-1 agonist, exenatide, was approved for the management of Type 2 diabetes mellitus (T2DM) [6]. In 2009, another GLP-1 agonist, liraglutide, reached the market, followed by the establishment of more GLP-1 agonists in diabetes management [7].

### Gastrointestinal Factors

Over the past century, many observations have been made supporting the presence of gastrointestinal (GI) substances that might increase insulin secretion. In 1906, Moore et al. suggested that the duodenal mucosa could potentially release a hormone that acts as an excitant for internal pancreatic secretions [8].

In 1959, the innovation of radioimmunoassay by Berson and Yalow helped quantify insulin secretion in humans [9]. Experiments undertaken in 1964 showed more insulin secretion with oral versus intravenous (IV) glucose administration despite identical glucose concentrations, an effect coined the “Incretin effect” [10].

In 1973, John Brown discovered the glucose-dependent insulinotropic polypeptide (GIP) employing peptide purification and protein sequencing, it was shown to have an incretin effect, increasing Insulin release triggered by glucose intake after oral administration [11]. However, preservation of the incretin effect after GIP removal has been demonstrated in animal GIP immunoneutralization experiments [12]. Studies in humans confirmed the lack of GIP effect on insulin secretion in patients with all DM types [13].

During the early 1970 s, the introduction of recombinant DNA (rDNA) technology enabled more precise protein amino acid sequencing by analyzing the nucleotide sequences of cloned recombinant cyclic DNA (cDNA) derived from messenger RNA (mRNA) [14]. Using this new technology, Habener et al. discovered the amino acid and gene sequences of proglucagon from anglerfish, which contained a stretch of amino acids resembling glucagon [14, 15]. Shortly thereafter, Graeme Bell et al. published the complete structure of mammalian and human proglucagon, which contained two glucagon-like structures called GLP-1 and GLP-2 [16]. However, there was uncertainty regarding the identification of the bioactive isoforms of Glucagon-like peptide 1 (GLP-1) that possessed a true insulinotropic effect.

Svetlana Mojsov first predicted the presence of GLP-1 isoforms [17]. Her subsequent work with Daniel Drucker using radioimmunoassay and chromatography identified a unique pattern of proglucagon-derived peptides in the gastrointestinal tract and pancreas, including more immunoreactive GLP-1 peptides in GI extracts [18]. Subsequent experiments showed that the active forms of GLP-1 were GLP-1(7–36 amide) and GLP-1(7–37) isoforms, increasing cAMP levels, insulin mRNA transcripts, promoting insulin secretion in the pancreases of pigs and rats, respectively [19]. In 1993, Nauck et al. showed that fasting glucose levels were completely normalized by GLP-1 infusion in T2DM patients [20]. This observation led to a study where GLP-1 was given to obese patients with T2DM and showed improved glycemic control with no side effects, establishing a proof-of-concept for GLP-1 agonists, which over time led to their use in the treatment of T2DM patients [21].

GLP-1 receptor agonists have been administered as subcutaneous injections. To overcome a potential barrier among patients favoring an alternative, oral formulations were developed. In 2019, oral semaglutide received approval as the first oral GLP-1 receptor agonist [22]. To improve gastrointestinal absorption, a carrier molecule of sodium N-(8-[2-hydroxybenzoyl] amino) caprylate (SNAC) was added to its chemical structure to overcome the challenges of peptide degradation and poor membrane permeability [23].

Looking ahead, the future of GLP-1RA delivery is poised to include oral, ultralong-acting injectables, implantable pumps, smart electronic devices, and noninvasive systems like sublingual or transdermal routes. These innovations aim to enhance bioavailability, reduce dosing frequency, and improve patient compliance [24].

#### A Pivotal Contribution from Regulatory Agencies To Relevant Clinical Observations

The requirements to assess the safety of new diabetes medications, including GLP-1R agonists, set forth by The United States Food and Drug Administration (FDA) were specified in the December 2008 guidance document titled “Guidance for Industry: Diabetes Mellitus — Evaluating Cardiovascular Risk in New Antidiabetic Therapies to Treat Type 2 Diabetes.” A draft of this document had been issued in March 2008 and the final document followed recommendations from the Endocrinologic and Metabolic Drugs Advisory Committee that convened on July 1 and 2, 2008 [25]. This guidance advised that companies should show that the risk of cardiovascular events in T2DM patients didn’t increase with the use of their new antidiabetic therapies (drugs or therapeutic biologics), a proactive response to concerns about the cardiovascular safety of some diabetes medications developed previously (https://www.fda.gov/regulatory-information/search-fda-guidance-documents/type-2-diabetes-mellitus-evaluating-safety-new-drugs-improving-glycemic-control-guidance-industry; accessed August 25, 2025). Similar recommendations were made by the European Medicines Agency (EMA) that required including major adverse cardiovascular events (MACE) as a primary endpoint (https://www.ema.europa.eu/en/clinical-investigation-medicinal-products-treatment-or-prevention-diabetes-mellitus-scientific-guideline).

These regulatory requirements led to the discovery of cardiovascular benefits in GLP-1R agonists, demonstrating the importance of due diligence and proactive safety monitoring. The agencies not only ensured safety but also facilitated the expansion of therapeutic indications for GLP-1R agonists, including obesity and cardiovascular risk reduction.

The cardiac benefits of GLP-1 agonists were not anticipated before outcome studies were performed for several reasons: (1) Historical Precedent-past experiences with other diabetes medications that unexpectedly had adverse cardiovascular effects made it less likely for researchers and clinicians to anticipate potential cardiovascular benefits without concrete evidence from large-scale trials; (2) Primary Focus on Glucose Control- GLP-1R agonists were developed to improve glycemic control in T2DM patients. The primary goals were to lower blood sugar levels and reduce the risk of diabetes-related complications; (3) Lack of Early Indicators- prior to the cardiovascular outcomes trials (CVOTs), there was no strong evidence or clear biological indicators suggesting that this class of drug would have a direct effect on cardiovascular health; and (4) Complexity of Cardiovascular Disease- cardiovascular disease is multifactorial and complex. It was not clear how a drug that primarily affects glucose metabolism might directly impact the occurrence of cardiovascular events.

The FDA released an updated guidance document for industry in March 2020 that focused on the safety of drugs for chronic use to improve glycemic control in T2DM patients ((https://www.fda.gov/regulatory-information/search-fda-guidance-documents/type-2-diabetes-mellitus-evaluating-safety-new-drugs-improving-glycemic-control-guidance-industry 2020 fda.gov; accessed August 25, 2025). An emphasis was placed on post-marketing surveillance informed by pre-marketing safety signals and on comorbid conditions that are common in T2DM patients, including: cardiovascular disease, chronic kidney disease, and older age.

The guidance document recommended that clinical trials include substantial representation of these populations to better reflect real-world use. Specifically, it called for safety data from at least 500 patients with stage 3 or 4 CKD, 600 with established CVD, and 600 aged over 65. This broadened inclusion criteria allowed for a more comprehensive understanding of drug effects in high-risk groups and supported the expansion of GLP-1R agonist indications beyond glycemic control to include cardiovascular and renal benefits.

The guidance emphasized post-marketing surveillance informed by pre-marketing safety signals, moving away from a rigid “one-size-fits-all” model. This flexibility enabled regulators to tailor safety monitoring based on actual risk profiles observed during drug development, facilitating the approval of drugs like GLP-1R agonists for broader therapeutic use, including in non-diabetic populations with Cardiovascular-Kidney-Metabolic (CKM) Syndrome.

In broader terms, the guidance exemplified how regulatory science can catalyze translational research, uncover unanticipated benefits, and accelerate innovation across therapeutic domains.

### Mechanisms of Action and Benefit

#### Biological, Physiological, and Molecular Considerations

Glucagon-like peptide 1 (GLP-1) is primarily secreted by enteroendocrine L-cells in the intestine, pancreatic α-cells, and the central nervous system (CNS) [26]. It is an incretin peptide hormone that participates in insulin secretion, contributing to bioenergetic pathways of cell survival [27]. After food intake, GLP-1 is rapidly released, enhancing insulin secretion in response to rising glucose levels [27].

**Fig. 1:**
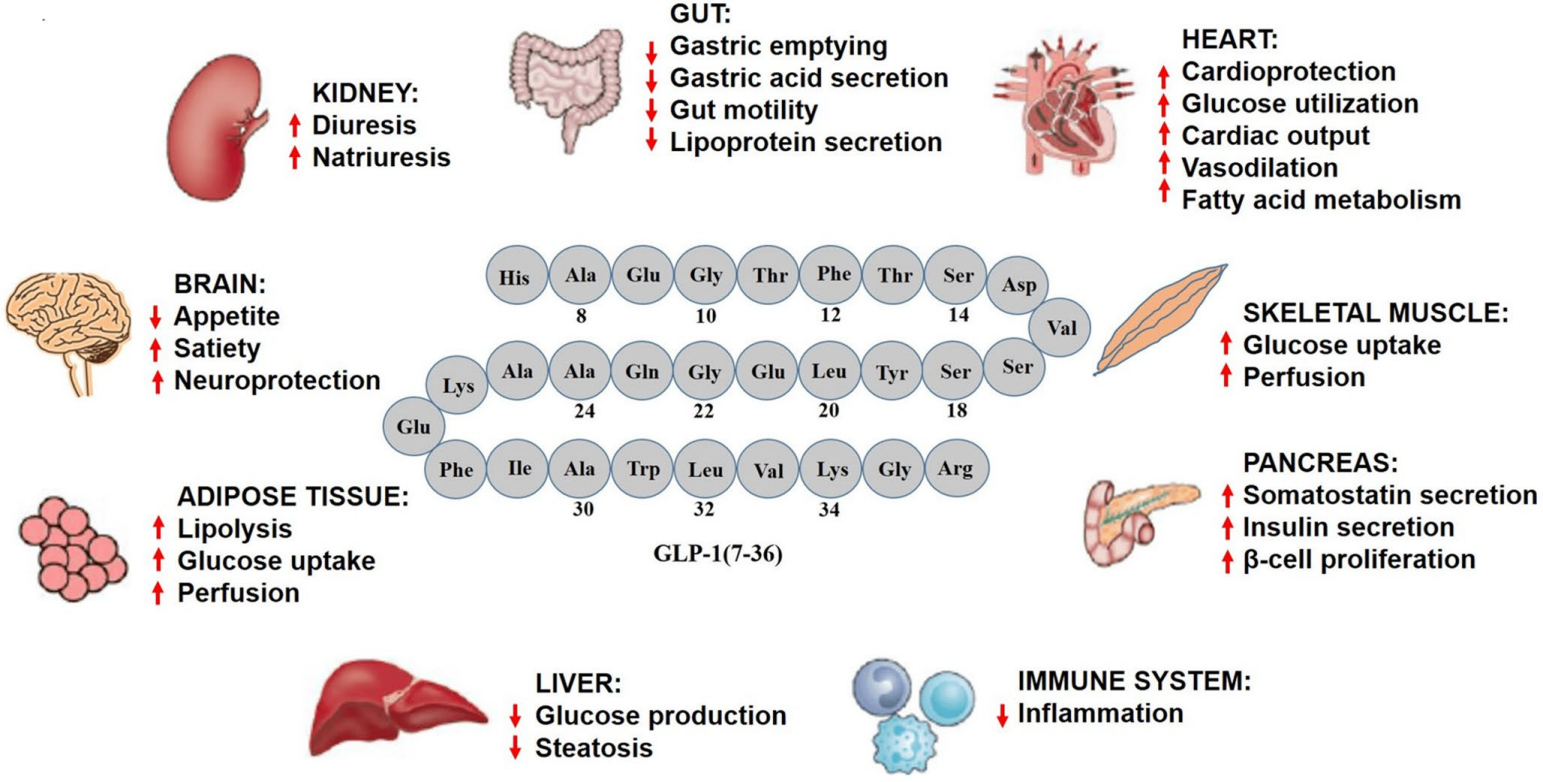
The multiple sites of Glucagon-like Peptide (GLP)−1 expression and their resulting biological and physiological effects (from Pharmaceuticals 2023; 16: 836)

GLP-1 receptor (GLP-1R) is class B of the G protein-coupled receptor (GPCR) and is primarily found in the CNS and pancreas. While its expression is most concentrated in these areas, it is also present in the lungs, heart, kidneys, digestive system, liver, skeletal muscles, and peripheral nervous system to a lesser extent [27, 28] (Fig. 1).

Once synthesized in by the ribosomes in the rough endoplasmic reticulum (RER), GLP-1R gets targeted to the smooth endoplasmic reticulum (SER) by a signal peptide located at the N-terminus of the receptor [29]. Cleavage of this signal peptide is crucial for GLP-1R expression on the cell surface; if cleavage is blocked, the receptor remains in the SER. Within its N-terminal domain, GLP-1R contains six conserved cysteine residues that form specific disulfide bonds between Cys46-Cys71, Cys62-Cys104, and Cys85-Cys126 [30]. Additionally, residues Asp67, Trp72, Pro86, Arg102, Gly108, and Trp110 are highly conserved across class B GPCRs, with Trp72 and Trp110 playing a vital role in agonist binding [29].

Once bound to GLP-1R, GLP-1 initiates a cascade of events, including the activation of membrane-bound adenylyl cyclase (AC) leading to cyclic adenosine monophosphate (cAMP) synthesis. cAMP formation initiates several downstream signaling pathways by activating downstream effector proteins such as protein kinase A (PKA) and exchange protein (Epac) activated by cAMP [31, 32].

### GLP-1R Gene

GLP-1R gene encodes the glucagon-like peptide 1 receptor, a protein essential for regulating glucose metabolism and promoting insulin secretion. It’s a 7-transmembrane protein that functions as a receptor for the GLP-1 hormone. GLP-1R gene expression has been demonstrated in many organs and organ systems, including the heart and cardiovascular system [30].

Transcription events related to the GLP-1R gene involve its activation by GLP-1 or its analogs, initiating a series of intracellular reactions. These reactions include increasing cAMP and intracellular calcium, activation of protein kinase A, and induction of gene transcription [33]. Furthermore, GLP-1R can also become internalized in response to GLP-1 and its analogs, indicating a complex regulatory mechanism that affects gene expression and protein activity [32].

### Evolutionary Biology

Upon nutrient ingestion enteroendocrine L-cells secrete GLP-1, which has a crucial role in glucose metabolism regulation via enhancing insulin secretion and inhibiting glucagon release. The evolutionary advantage of this system is to ensure efficient nutrient utilization and energy storage, which are critical for survival during periods of food scarcity [34, 35].

The cardiovascular effects of GLP-1 can be seen as an extension of its role in maintaining metabolic homeostasis. By improving cardiovascular function, GLP-1 helps to ensure that nutrients and oxygen are efficiently delivered to tissues, which is essential for overall metabolic health [36].

### Embryology

The biological and physiological interface between the gastrointestinal (GI) tract and the cardiovascular system can be explained from an embryological perspective by examining the co-development of these systems during organogenesis.

During embryogenesis, the heart and the GI tract develop in proximity and share common signaling pathways and structural components. The heart originates from the mesoderm, while the GI tract arises from the endoderm. The interaction between these germ layers is crucial for the proper development of both systems.

Research has shown that the endoderm has a significant role in heart morphogenesis. For example, the endoderm provides essential paracrine signals that influence cardiac specification and differentiation. This is evident in the co-development of heart and digestive system tissues within human induced pluripotent stem cell (iPSC)-derived organoids, where the presence of endodermal tissue promotes the maturation of cardiac tissues [37].

In addition, the serosal mesothelium, which covers the gut, is a major source of gut vasculature smooth muscle cells. This mesothelium undergoes epithelial-mesenchymal transition (EMT) and plays a role vascular smooth muscle cells development, highlighting a conserved mechanism in blood vessel development for coelomic organs, including the heart and gut [38].

During heart tube assembly, the endoderm mechanical role is critical as it contracts, pulling the heart fields towards the midline to facilitate heart tube formation. This mechanical interaction underscores the importance of the endoderm in guiding the morphogenesis of the heart [39].

### Gastrointestinal- Cardiovascular System Signaling

The gut-heart axis involves bidirectional communication where gut microbiota and their metabolites, such as short-chain fatty acids (SCFAs) and trimethylamine-N-oxide (TMAO), influence cardiovascular health. SCFAs have protective cardiovascular effects, while TMAO and lipopolysaccharides (LPS) from gut dysbiosis exacerbate cardiovascular diseases by promoting inflammation and endothelial dysfunction [40].

Vasoactive Intestinal Peptide (VIP), another gut-derived peptide, acts as a potent vasodilator and has positive inotropic effects on the heart. It increases coronary blood flow and reduces vascular resistance, contributing to improved cardiac function and protection against ischemic injury [41].

These interactions highlight the complex and integrated role of gut-derived peptides in modulating cardiovascular function, emphasizing the therapeutic potential of targeting these pathways in cardiovascular diseases as captured below under gaps and new areas of investigation.

### Glucagon-Like Peptide-1 (GLP-1) and its Cardiovascular Effects

Over the past thirty years, researchers have made significant strides in understanding the gut-derived hormones impact on cardiovascular health. Among these hormones, GLP-1 has emerged as a pivotal role in cardiovascular disease and health.

GLP-1R agonists exert their cardiovascular effects through multiple pathways.

### Positive Inotropic and Chronotropic Effects

GLP-1 exerts positive inotropic and chronotropic effects on the heart. By enhancing myocardial contractility, GLP-1 contributes to efficient cardiac function [42].

### Preserve Left Ventricular Structure and Function

GLP-1 maintains left ventricular integrity through both direct and indirect mechanisms. It contributes to cardiac functional and structural well-being. These effects particularly are relevant in preventing adverse remodeling and heart failure progression [43].

### Pro-Survival and Energy Utilization

GLP-1 activates pro-survival kinases within cardiac cells. These kinases promote cell survival, potentially protecting against ischemic injury and oxidative stress. Furthermore, GLP-1 enhances energy utilization, optimizing cardiac metabolism [44].

### Immune Modulating Effects

Induced human regulatory T cells (iTregs) express a functional GLP-1R, which increases intracellular cAMP levels upon stimulation with a GLP-1R agonist. This suggests that GLP-1R signaling in iTregs may mediate GLP-1R agonists anti-inflammatory effects [45]. A subset of CD8 T cells also express GLP-1R when exhausted. These GLP-1R-positive CD8 T cells has a role in the alloimmune response, and their signaling through GLP-1R acts as a negative costimulatory mechanism. This signaling can prolong allograft survival and mitigate alloimmune responses [46].

Proinflammatory cytokine expression in the myocardium has been shown to be reduced by GLP-1R agonists, which directly protects the heart against oxidative stress and inflammation. These combined effects on immune cells and direct myocardial protection underscore the multifaceted cardioprotective benefits of GLP-1R agonists [47].

### Inflammation Attenuating Effects

Evidence suggests that inflammation in organs such as the liver, kidneys, and heart can be reduced by GLP-1R agonists [48]. In addition, by minimizing inflammation and preventing apoptosis they have a cellular protective role in the nervous and cardiovascular systems [49]. GLP-1R agonists regulate various molecular pro-inflammatory mediators, including oxidative stress, cytokine production, immune cell recruitment, glucotoxicity, and lipotoxicity ([50]). Employing preclinical, ex vivo, in vivo, and in vitro model systems investigators determined that GLP-1 agonists exert anti-inflammatory and immune modulating effects through their effects on macrophages (reduced interleukin [IL] 1β, IL-6, tumor necrosis factor [TNF]α, and C-X-C motif chemokine ligand [CXCL]), T and B lymphocytes (reduced cluster of differentiation [CD] 4 cells), monocytes (increased IL-10), mononuclear cells (decreased monocyte chemoattractant protein [MCP]−1, cell death protein [PD],, CD4 and CD8 T cells) [51–55].

Shiraki and colleagues determined that GLP-1 agonists anti-inflammatory effect were particularly robust on vascular endothelial cells [56]. GLP-1 agonists has protective effects against inflammation and oxidative stress on human endothelial cells, including those injured by inflammatory mediators like tumor necrosis factor (TNF)-α [57]. The inhibition of protein kinase C (PKC), NADPH oxidase, and NF-ҡB signaling exhibited anti-oxidative and anti-inflammatory effects in endothelial cells, accompanied by the upregulation of protective anti-oxidative enzymes [57]. Favorable effects were also demonstrated by Krasner and colleagues [58].

### Anti-atherosclerosis Effects

Atherosclerosis, a chronic inflammatory disorder of the arterial wall, involves several key contributors to its initiation and progression that can be favorably altered or attenuated by GLP-1 agonists. Low density lipoprotein (LDL) cholesterol (C) and oxidized (ox) LDL are involved in atherogenesis, including the initial stage of endothelial cell injury and inflammation. GLP-1 agonists reduce LDL-C and ameliorate ox-LDL’s proinflammatory injurious effects on vascular endothelial cells that include monocyte binding [59]. Yue and colleagues showed that the GLP-1 agonist, liraglutide, reduced the transcriptional factor KLF2 and the rescued ox-LDL induced reduction of mitogen-activated protein kinase (MAPK) kinase extracellular signal regulated kinase 5 (ERK5) phosphorylation, and blockage of ERK5 activity on KLF2 expression [60]. Liraglutide also reduced endothelial tight junction protein barriers, ameliorated ox-LDL induced endothelial monolayer permeability, and inhibited ox-LDL induced expression of vascular adhesion molecules (E-selectin and vascular cell adhesion molecule- 1), preventing ox-LDL induced monocyte adhesion to endothelial cells [57].

GLP-1R are present in human coronary artery endothelial cells [61]. There is interest in GLP-1 agonists and their ability to either reduce atherosclerotic plaque burden or the potential for plaque disruption- recognized as a proximate cause of arterial thrombotic events. There are supportive clinical trials in T2DM patients of GLP-1 agonist mediated reduction in plaque burden, size and composition [62].

Despite a growing body of work, the precise mechanism(s) whereby GLP-1R agonists achieve anti-atherogenic effects is an area of much needed investigation.

### Vasodilatory Effects

GLP-1 agonists exert vasodilatory properties in several vascular beds, ranging from the aorta to the coronary, mesenteric and renal arteries. The mechanism(s) represents a combination of direct and indirect effects on smooth muscle and endothelial cells. Selley and colleagues reported that GLP-1 associated vasodilation was mediated through the glucagon receptor [63]. Koska et al. found that increased postprandial endothelial function (EF) was independent of reductions in plasma glucose and triglycerides. The GLP-1 agonist, exenatide, increased fasting EF via endothelial nitric oxide (NO) synthase (eNOS) activation, nitric oxide (NO) production in endothelial cells, induced dose-dependent vasorelaxation, and reduced high-glucose or lipid-induced endothelial dysfunction in arterioles ex vivo [64].

GLP-1R agonists effect(s) on vascular smooth muscle cells are equally important to those on endothelial cells with several favorable responses being reported, including enhanced differentiation, attenuated response to reactive oxygen species, reduced vascular remodeling through improved mitochondrial activity and dynamics, and the prevention of aberrant migration [65].

#### Microcirculation

Animal studies suggest that GLP-1R agonists enhance microvascular function and myocardial perfusion. Sukumaran and colleagues treated Zucker lean and obese rates with either vehicle or the GLP-1 agonist liraglutide (LIRA) for 8 weeks [66]. Synchrotron contrast microangiography was employed to assess coronary arterial vessel function (internal diameter: 50–350) in vivo in anesthetized rats. Myocardial gene and protein expression levels of vasoactive factors, inflammatory markers, oxidative stress and remodeling indicators were analyzed using real-time PCR and Western blotting. Compared to vehicle-treated rats, those receiving LIRA exhibited significant improvement in acetylcholine-mediated vasodilation within small arteries and arterioles (< 150 μm diameter). LIRA did not significantly alter soluble guanylyl cyclase or endothelial nitric oxide synthase (eNOS) mRNA levels, nor total myocardial eNOS protein expression. However, LIRA led to a notable downregulation of Nox-1 mRNA (*p* = 0.030) and a reduction in ET-1 protein expression (*p* = 0.044). Moreover, LIRA significantly suppressed the expression of proinflammatory and profibrotic biomarkers, including NF-κB, CD68, IL-1β, TGF-β1, osteopontin, and nitrotyrosine, in comparison to vehicle-treated rats [66]. There have been equally favorable observations in healthy volunteers with obesity and patients with either diabetes, stable coronary artery disease or unstable coronary artery disease. In each group microvascular blood flow velocity, blood volume, and myocardial perfusion were increased and improved, respectively, to variable degrees [67].

### Favorable Metabolic Effects

GLP-1R agonists has shown to reduce the effects of several atherosclerotic cardiovascular disease (ASCVD) metabolic risk factors including diabetes mellitus, hyperlipidemia, overweight and obesity, and hepatic steatosis [68].

### Anti-hypertensive Effects

Essential hypertension is ubiquitous worldwide and contributes substantially to cardiovascular disease-associated morbidity and mortality. GLP-1 agonists exert anti-hypertensive effects compared to placebo [69], reducing systolic blood pressure by 2–3 mmHg. There are several mechanisms that include vasodilation, natriuresis, and decreased sympathetic activity [70].

### Neurohumoral Effects

In the central nervous system, GLP-1Rs are highly expressed in regions involved in metabolic regulation, appetite, and autonomic control. Key sites include the hypothalamic nuclei (arcuate, paraventricular, dorsomedial), area postrema, nucleus of the solitary tract (NTS), dorsal motor nucleus of the vagus nerve, amygdala, lateral septum, hippocampus, and cortex. These receptors are present on both neuronal cell bodies and axonal projections and are found in GABAergic and glutamatergic neurons depending on the region [71].

In the peripheral nervous system, GLP-1Rs are expressed on vagal afferent neurons, enteric neurons, dorsal root ganglia, and nerves innervating metabolically active tissues such as adipose tissue and the gastrointestinal tract. Vagal neurons are a major site for peripheral GLP-1 action, mediating gut-brain communication for the regulation of feeding, gastric emptying, and glucose metabolism [72]. GLP-1Rs are also present on Schwann cells and sensory, motor, and enteric neurons, implicating them in peripheral neuropathy and metabolic disease modulation [73]. GLP-1Rs agonists exert neuro-humoral actions by stimulating insulin secretion and suppressing glucagon release in a glucose-dependent manner, delaying gastric emptying, and reducing appetite via central nervous system pathways. These agents act on GLP-1Rs distributed in pancreatic, gastrointestinal, cardiovascular, hepatic, and neural tissues, mediating both peripheral and central effects.

Neuro-humoral actions include modulation of hypothalamic appetite centers, leading to increased satiety and reduced caloric intake, as well as slowing of gastric emptying, which further contributes to appetite suppression and glycemic control [74]. GLP-1R agonists also demonstrate neuroprotective effects, including reduction of neuroinflammation, oxidative stress, and neuronal apoptosis, with emerging evidence for benefit in neurodegenerative diseases such as Parkinson’s and Alzheimer’s disease [75, 76].

### Vascular Aging Effects

Vascular aging, marked by structural and functional changes in the vascular wall, is a hallmark of the aging and is strongly linked to cardiovascular mortality and age-related vascular disorders development [77]. Aging is a diverse and multifaceted process as different organs within an individual undergo distinct aging trajectories influenced by genetic, environmental, and lifestyle factors [78].

Vascular aging involves several key components that contribute to the decline in vascular health over time. Arteries and veins lose their elasticity, leading to increased vascular stiffness, which can result in higher blood pressure and a higher risk of cardiovascular diseases. Additionally, vascular endothelium becomes less effective at regulating blood flow and preventing clot formation, leading to endothelial dysfunction, increasing the risk of atherosclerosis, thrombosis, and other vascular diseases. An imbalance between the reactive oxygen species (ROS) production and the body’s detoxification ability cause oxidative stress, damaging cells and tissues and contributing to vascular aging. Chronic low-grade inflammation also has a major role in vascular aging, potentially leading to various age-related diseases, including cardiovascular diseases.

Other important determinants of vascular aging include genes that are predisposed to lipid and metabolic conditions, epigenetic modifications, psychosocial stress, and environmental exposures to pollutants [77, 79].

Exenatide, a first-generation GLP-1 agonist, mitigated vascular aging induced by a high-fat diet in ApoE-/- mice by regulating inflammation and oxidative stress responses [55]. It also lessened ischemia-reperfusion injury induced endothelial dysfunction in humans through the activation of K ATP channels [80].

GLP-1 receptor activation increases DNA repair by enhancement of apurinic/apyrimidinic endonuclease 1 (APE1) expression [81]. Additionally, GLP-1 mitigates H2O2-induced senescence, regulates the antioxidant defense system, reduces cellular senescence, and DNA damage triggered by recurring oxidative stress [82].

#### Organ System Signaling and Vascular Aging

Signaling or “crosstalk” is common across almost all organ systems. For example, the heart and kidneys have a complex and interconnected relationship. This interaction is crucial for maintaining overall cardiovascular and renal health. It is also important for understanding the effect(s) of drug therapy that targets pathways and receptors that are shared between organs. The renin-angiotensin-aldosterone system (RAAS) has a significant role in regulating blood pressure, blood volume, and response to alterations in renal or myocardial performance. The kidneys are also involved with sympathetic nervous system (SNS) activation and tone, inflammation, and immune regulation [83].

Accelerated kidney aging promotes diabetic kidney disease. Kidney aging in diabetic individuals is the cumulative effect of cellular senescence, advanced glycation end product accumulation, RAAS dysfunction, inflammation, and oxidative stress. The molecular and structural/functional changes translate to (1) Tubular atrophy, podocyte hypertrophy, glomerulosclerosis, and microvascular rarefaction and (2) glomerular basement membrane thickening, nephrosclerosis, and extracellular matrix accumulation, respectively [84].

GLP-1R agonists have favorable long-term effects on renal function, particularly in patients with diabetic kidney disease (DKD). Decreased aging, improved performance that are attributable to improved blood pressure control, improved glycemic control, weight loss, increased renal blood flow, restoration of normal glomerular hemodynamics, enhanced autoregulation, and prevention of oxidative injury are potential contributors to reducing cardiovascular events through the kidney-cardiovascular axis [85].

The liver and cardiovascular system engage in complex crosstalk through various mechanisms to maintain homeostasis and contribute to disease progression when dysregulated. Several examples of Metabolic Regulation: The liver has a central role in lipid and glucose metabolism, which directly impacts cardiovascular health. Dysregulation in liver metabolism, such as in nonalcoholic fatty liver disease (NAFLD), can increase cardiovascular risk. Chronic inflammation is a common link between liver and cardiovascular diseases. Inflammatory cytokines produced by the liver can affect cardiovascular function, and vice versa. Both liver and cardiovascular diseases involve fibrotic processes. For instance, liver fibrosis can lead to systemic inflammation and cardiovascular remodeling. Moreover, the liver produces many hormones and growth factors that influence cardiovascular function. For example, coagulation factors produced by the liver can affect heart function [86].

GLP-1R agonists by reducing de novo lipogenesis and promoting fat oxidation leads to liver fat reduction and liver function improvement in NAFLD patients. Furthermore, studies have shown that GLP-1 agonists reduce liver-associated mortality in T2DM patients [87].

### Antithrombotic Effects

GLP-1 agonists reduce cardiovascular events through several mechanisms previously summarized to include improved glycemic control, weight loss, lowering blood pressure, lipid lowering, reduced atherogenesis, attenuated vascular inflammation, improved endothelial cell function, vasodilation, and direct cardiovascular protective effects [88, 89]. The direct cardiovascular effects represent a combination of platelet inhibition, augmented fibrinolytic responses, and attenuated thrombogenesis that collectively address not only the prothrombotic phenotypes of DM mellitus, obesity, and chronic kidney disease but a novel prevention approach beyond traditional risk factors for myocardial infarction, ischemic stroke, and cardiovascular death [90, 91].

#### Megakaryocytes and Platelets

From a human megakaryocyte cell line (MEG-01), Cameron-Vendrig and colleagues cloned the complete GLP-1R mRNA and discovered that GLP-1R expression levels in MEG-01 cells were higher than those found in human lung tissue [92]. Incubation with GLP-1 and the GLP-1R agonist, exenatide, triggered a cAMP response in MEG-01 cells, with exenatide significantly reducing platelet aggregation induced by thrombin, ADP, and collagen. Also, exenatide suppressed thrombus formation under flow conditions in ex vivo perfusion chambers using both human and mouse whole blood. In a mouse cremaster artery laser injury model, a single intravenous dose of exenatide inhibited thrombus formation in both normoglycemic and hyperglycemic mice. Thrombus formation was more pronounced in mice transplanted with bone marrow deficient in functional GLP-1R (Glp1r−/−) compared to those with wild-type bone marrow, with these effects being dependent on nitric oxide [57].

**Fig. 2:**
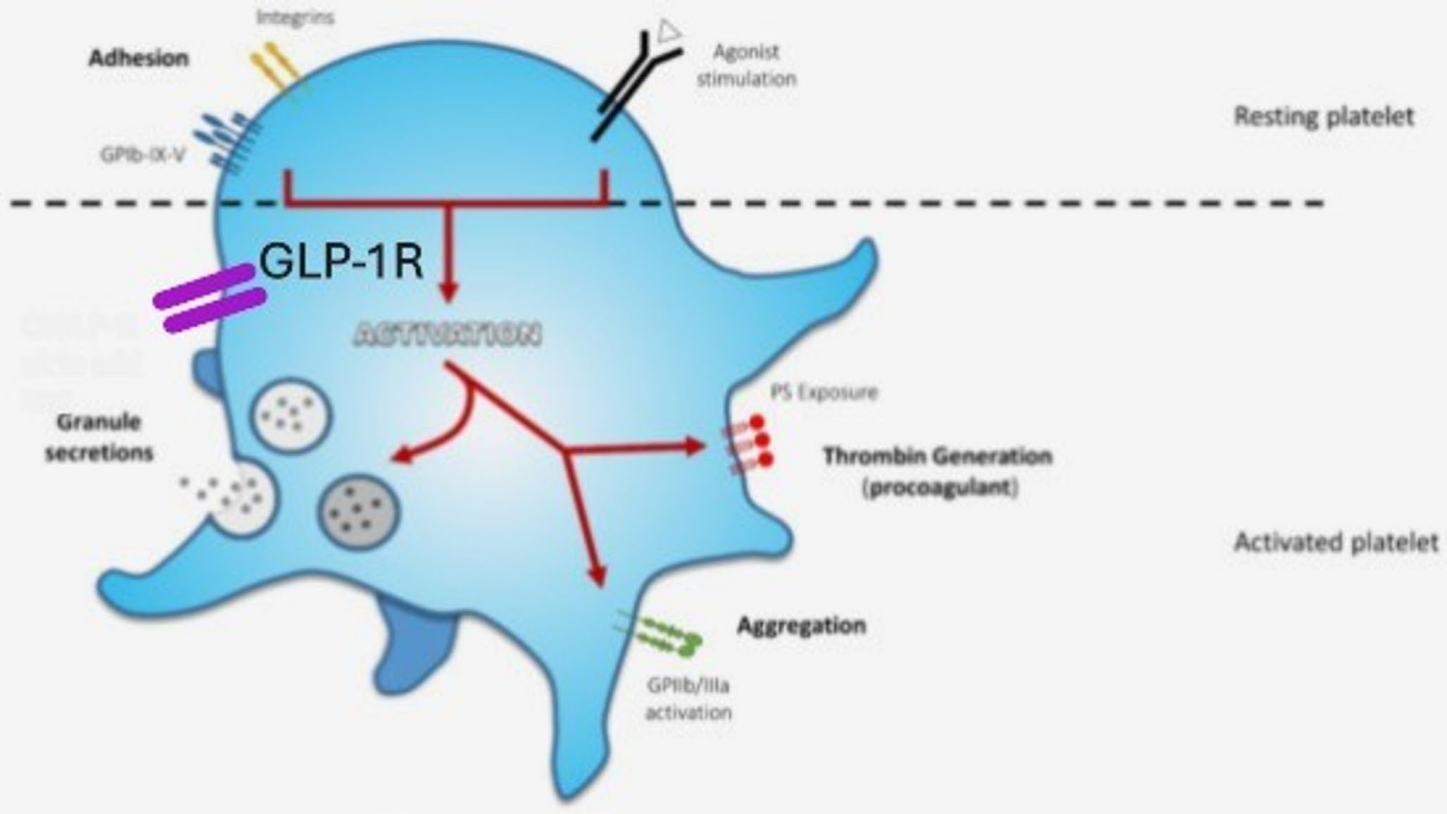
Principal Activation Endpoints During Platelet Activation. At first, platelet receptors interact with adhesive agonists exposed at the site of lesion: von Willebrand factor (VWF) binds to glycoprotein (GP) Ib-IX-V complex and collagen interacts with integrin α_2_β_1_ for adhesion and GPVI to mediate platelet activation. These first interactions initiate platelet response. Soluble agonists released by either activated platelets or injured tissue amplify platelet response and activation. These agonists induce proper receptor activation and their signaling converge to activate a core set of intracellular signaling pathways leading to various activation endpoints, such as shape change and formation of pseudopodia, secretion of α-granule and dense granule content, activation of GPIIb/IIIa sustaining platelet aggregation (from J Clin Med 2021;10: 894). Glucagon-like peptide (GLP)−1 agonists bind to platelets through a distinct receptor (GLP-1R) and increase cyclic AMP (adenosine monophosphate) decreasing platelet aggregation in response to thrombin, ADP (adenosine diphosphate) and collagen. Receptor activation also inhibits ADP- mediated platelet activation and aggregation through down-stream mechanisms (see text)

GLP-1Rs are also expressed in platelets. When GLP-1 or GLP-1R agonists bind to these receptors, they trigger a cAMP response, which inhibits platelet aggregation. Specifically, GLP-1 receptor activation increases intracellular cAMP levels, which in turn activates protein kinase A (PKA). PKA phosphorylates and inhibits key proteins involved in platelet activation, such as phosphodiesterase 3 A (PDE3A), which normally degrades cAMP. Moreover, ADP binds to its receptors P2Y1 and P2Y12 on platelets, promoting platelet activation and aggregation. GLP-1 receptor activation leads to increased cAMP, which inhibits the signaling pathways downstream of these ADP receptors, thereby reducing platelet activation [92]. In addition, agonist binding increases NO-antiaggregating effects, cGMP/PKG/VASP pathway activation, reduces of PI3-K/Akt and MAPK/ERK-2 pathways activation, and decreases arachidonic acid (AA)-induced oxidative stress [93, 94] (Fig. 2).

GLP-1R activation decreases thrombus formation under flow conditions. This has been demonstrated in both ex vivo perfusion chambers with human and mouse whole blood and in vivo models, such as the mouse cremaster artery laser injury model [92].

These effects do not significantly differ between various GLP-1R agonists. Both native GLP-1 and its analogs, including liraglutide and exenatide, exhibit similar inhibitory effects on platelet activation formation and thrombus. The antithrombotic properties are consistent across different agents, suggesting a class effect rather than agent-specific differences [94].

#### Fibrinolysis

Fibrinolytic activity is crucial for vascular health. It maintains physiological hemostasis, prevents non-hemostatic thrombus formation, facilitates wound healing, and regulates inflammation on the endothelial cell surface [95]. GLP-1 agonists can reduce plasminogen activator inhibitor (PAI)−1 levels, thereby augmenting fibrinolytic potential [96, 97].

#### Prothrombotic Pathways

Deng et al. employed network pharmacology to identify the mechanism(s) and pathways underlying reduced myocardial infarction rates in DM patients receiving GLP-1 agonists. Gene Ontology (GO) and Kyoto Encyclopedia of Genes and Genes (KEGG) enrichment analyses were conducted, utilizing the STRING database to construct the protein-protein interaction (PPI) network. Cytoscape was used to pinpoint core targets, transcription factors, and modules. The GO and KEGG analyses highlighted enrichment terms predominantly associated with the extracellular matrix (ECM), coagulation, RAS, and endopeptidase. Notably, four of the seven core targets were linked to the regulation of ECM deposition and remodeling, processes relevant to atherosclerotic plaque formation. Additional significant enrichment categories included coagulation, complement activation, and platelets [98].

The antithrombotic properties of GLP-1R agonists are complementary to those offered by platelet antagonists (aspirin, clopidogrel) and are not associated with an increased bleeding risks [99, 100].

While GLP-1R agonists have demonstrated cardiovascular benefits in clinical trials, including reduced rates of major adverse cardiovascular events (MACE) in T2DM patients, their antithrombotic efficacy is generally considered less potent than that of traditional antithrombotic agents. The American Heart Association and the Heart Failure Society of America note that GLP-1R agonists can decrease systemic inflammation and platelet aggregation, contributing to their cardiovascular benefits [101].

### Science and Clinical Translation

The diverse and interconnected mechanisms of action of GLP-1 receptor agonists—spanning cardiac, vascular, endothelial, platelet, and broad intracellular signaling pathways—establish a robust scientific foundation and a clear roadmap for their investigation, clinical trial design, and therapeutic use in medicine. These agents exert beneficial effects on myocardial contractility, vascular tone, endothelial integrity, and platelet aggregation, while also modulating inflammation, oxidative stress, and metabolic homeostasis at the cellular and molecular levels. Their ability to engage in multiple organ systems through biologically conserved pathways has not only validated their initial use in glycemic control but also catalyzed their expansion into cardiovascular, renal, and metabolic domains. The depth and breadth of mechanistic insight continue to inspire new research directions and clinical applications, fueling enthusiasm for broader use and the realization of additional therapeutic benefits across diverse patient populations.

### Clinical Trial-based Evidence of Benefit

Research has shown that GLP-1R agonists play a significant role in reducing the likelihood of MACE, such as MI, cardiovascular death, and stroke [102]. A summary of clinical trials to include drug, dosing strategy, sample size, primary outcome measurements, risk reduction, date of publication, and conclusions is found in Table 1 [103–112]. These benefits are especially significant for patients with established cardiovascular disease or several cardiovascular risk factors.

**Table 1:** Clinical trials of GLP-1 agonists

Trial	Yr	Author	NCT Number	Drug/Dose	Trial Hypothesis	Result	Conclusion
STARDUST Trial (31)	2024	Marc A Pfeffer	NCT04881110	Liraglutide 1.8 mg SubQ Weekly	The effect of liraglutide on peripheral perfusion was measured as peripheral transcutaneous oxygen pressure (TcPo2) in individuals with type 2 diabetes and PAD.	*N* = 55 participants were randomized (27 to the liraglutide group and 28 to the control group). Transcutaneous PO2 increased over time in both groups, with significant differences favoring the liraglutide group after 6 months. The 10% increase in TcPO2 occurred in 24 participants (89%) in the liraglutide group and 13 (46%) in the control group. Individuals in the liraglutide group had a significant reduction of C-reactive protein, urinary albumin to creatinine ratio, and improvement of 6-minute walking distance.	In people with type 2 diabetes and PAD, liraglutide increased peripheral perfusion detected by TcPo2 measurement during 6 months of treatment. These results support the use of liraglutide to prevent the clinical progression of PAD in individuals with type 2 diabetes.
SELECT Trial (30)	2023	A Michael Lincoff	NCT03574597	Semaglutide 2.4 mg SubQ weekly	Testing whether the addition of semaglutide to standard care would be superior to placebo in reducing the risk of major adverse cardiovascular events among patients with overweight or obesity and preexisting cardiovascular disease who did not have diabetes	17,604 patients were enrolled; 8803 were assigned to receive semaglutide and 8801 to receive a placebo for 34 months, followed by 39.8 months. Primary cardiovascular end-point events occurred in 569 of the 8803 patients (6.5%) in the semaglutide group and 701 of the 8801 patients (8.0%) in the placebo group.	In patients with preexisting cardiovascular disease and overweight or obese but without diabetes, weekly subcutaneous semaglutide at a dose of 2.4 mg was superior to placebo in reducing the incidence of death from cardiovascular causes, nonfatal myocardial infarction, or nonfatal stroke at a mean follow-up of 39.8 months.
AMPLITUDE-O Trial (29)	2023	Hertzel C Gerstein	NCT03496298	Efpeglenatide 4 mg, 6 mg weekly	Assessment of a dose-response relationship of Efpeglenatide On major adverse cardiovascular events (nonfatal myocardial infarction, nonfatal stroke, or death from cardiovascular or unknown causes)	A clear dose-response was noted for all primary and secondary outcomes, major adverse cardiovascular outcomes occurred in *N* = 125 participants assigned to placebo, 84 participants assigned to 6 mg of efpeglenatide, and 105 assigned to 4 mg of efpeglenatide.	The graded salutary relationship between efpeglenatide dose and cardiovascular outcomes suggests that titrating efpeglenatide and potentially other glucagon-like peptide-1 receptor agonists to high doses may maximize their cardiovascular and renal benefits.
PIONEER 6 Trial (28)	2019	Mansoor Husain	NCT02692716	Semaglutide 14 mg, Oral daily	rule out an excess in cardiovascular risk with oral semaglutide among patients with type 2 diabetes	*N* = 3183 patients were assigned to oral semaglutide Vs placebo, followed by 15.9 months. 2695 patients were > 50 and had cardiovascular disease or CKD. Major adverse cardiovascular events happened in 3.8% of the treatment group vs. 4.8% of the placebo group.	The cardiovascular risk profile of oral semaglutide was not inferior to that of placebo.
Rewind Trial (27)	2019	Hertzel C Gerstein	NCT01394952	dulaglutide (1.5 mg), weekly SubQ	The effect of dulaglutide in reducing cardiovascular events in people with type 2 diabetes who have cardiovascular disease or are at high risk for it (aged at least 50 years with type 2 diabetes who had either a previous cardiovascular event or cardiovascular risk factors)	*N* = 9901 participants followed for 5.4 years. The primary outcome was the first occurrence of the composite endpoint of non-fatal myocardial infarction, non-fatal stroke, or death from cardiovascular causes (including unknown causes) occurred in 12% participants at an incidence rate of 2·4 per 100 person-years in the dulaglutide group and 13·4% participants at an incidence rate of 2·7 per 100 person-years in the placebo group. No difference in all-cause mortality.	Dulaglutide could be considered for the management of glycemic control in middle-aged and older people with type 2 diabetes with either previous cardiovascular disease or cardiovascular risk factors.
Harmony Outcomes (26)	2018	Adrian F Hernandez	NCT02465515	Albiglutide (30–50 mg) weekly, SubQ	Determine if albiglutide reduces the occurrence of major adverse cardiovascular events (such as cardiovascular death, non-fatal myocardial infarction, and non-fatal stroke) in patients with type 2 diabetes and cardiovascular disease.	In *N* = 9463 participants, the primary composite outcome (MACE) occurred in 7% of patients at an incidence rate of 4·6 events per 100 person-years in the albiglutide group and in 9% of patients at an incidence rate of 5·9 events per 100 person-years in the placebo group with relative risk reduction for MACE by 22%.	In patients with type 2 diabetes and cardiovascular disease, albiglutide was superior to placebo for major adverse cardiovascular events. Evidence-based glucagon-like peptide 1 receptor agonists should therefore be considered as part of a comprehensive strategy to reduce the risk of cardiovascular events in patients with type 2 diabetes.
EXSCEL Trial (25)	2017	Rury R. Holman	NCT01144338	extended-release exenatide, 2 mg weekly subQ	Effect of Exenatide on the incidence of major adverse cardiovascular events (MACE) vs. placebo	In *N* = 14,752 participants, exenatide was non-inferior to the placebo for safety but was not superior to the placebo for efficacy, the incidence of MACE did not differ in the two groups.	Among patients with type 2 diabetes with or without previous cardiovascular disease, the incidence of major adverse cardiovascular events did not differ significantly between patients who received exenatide and those who received placebo.
Leader Trial (22)	2016	Steven P Marso	NCT01179048	Liraglutide 1.8 mg or the maximum tolerated dose, daily SubQ	liraglutide would be non-inferior to placebo about major cardiovascular events (MACE) in patients with type 2 diabetes mellitus who are at high risk for cardiovascular events.	*N* = 9340 patients followed up for 3.8 years. The primary outcome was lower in the liraglutide group 13% than in the placebo 14.9%. Fewer patients died from cardiovascular causes in the liraglutide group 4.7% than in the placebo group 6.0%, The rate of death from any cause was lower in the liraglutide group 8.2% than in the placebo group 9.6%	In the time-to-event analysis, the rate of the first occurrence of death from cardiovascular causes, nonfatal myocardial infarction, or nonfatal stroke among patients with type 2 diabetes mellitus was lower with liraglutide than with placebo.
Sustain 6 Trail (24)	2016	Steven P Marso	NCT01720446	Semaglutide 0.5–1.0.5.0 mg weekly, subQ	semaglutide would be non-inferior to placebo for the primary outcome (first occurrence of cardiovascular death, nonfatal myocardial infarction, or nonfatal stroke) in patients with DM type 2.	*N* = 3297, 83% had established cardiovascular disease, chronic kidney disease, or both. The primary outcome occurred in 6.6% in the semaglutide group and 8.9% in the placebo Group. Nonfatal myocardial infarction occurred in 2.9% of the patients receiving semaglutide and 3.9% of those receiving placebo. Nonfatal stroke occurred in 1.6% and 2.7%, respectively. Rates of death from cardiovascular causes were similar in the two groups.	In patients with type 2 diabetes who were at high cardiovascular risk, the rate of cardiovascular death, nonfatal myocardial infarction, or nonfatal stroke was significantly lower among patients receiving semaglutide than among those receiving placebo. This outcome confirmed the noninferiority of semaglutide.
ELIXA Trial (23)	2015	Marc A Pfeffer	NCT01147250	Lixisenatide (starting dose 10 mg daily, target dose 20 mg daily) subQ	assess the effects of lixisenatide on cardiovascular morbidity and mortality in patients with Dm type 2 and acute coronary syndrome (in patients who had a myocardial infarction or who had been hospitalized for unstable angina within 180 days in addition to standard care.	6068 patients followed for 25 months, a primary end-point event occurred in 13.4% in the lixisenatide group and 13.2% in the placebo group. There were no significant between-group differences in the rate of hospitalization for heart failure or the rate of death.	In patients with type 2 diabetes and a recent acute coronary syndrome, the addition of lixisenatide to usual care did not significantly alter the rate of major cardiovascular events or other serious adverse events.

### GLP-1R Agonist Use in Clinical Practice

In both Europe and the United States, GLP-1R agonists are approved for a range of metabolic and cardiovascular conditions. Their principal indication remains for the management of T2DM, with agents such as exenatide, liraglutide, dulaglutide, semaglutide, Lixisenatide, and tirzepatide approved to enhance glycemic control. Certain agents, including semaglutide and liraglutide, are additionally indicated for long‑term weight management in individuals with obesity or overweight, typically in the presence of at least one weight‑related comorbidity. In addition, semaglutide, Liraglutide, and dulaglutide have indications for reducing cardiovascular risk in patients with T2DM and established atherosclerotic cardiovascular disease [113–118]. In the United States, Tirzepatide has recently gained approval for treating moderate to severe obstructive sleep apnea in adults with obesity [119] as well as semaglutide use for metabolic dysfunction–associated steatohepatitis (MASH) with moderate‑to‑advanced fibrosis [120]. These approvals reflect the expanding therapeutic role of GLP-1R agonists beyond glycemic control into broader cardiometabolic and respiratory domains Table 2.

**Table 2:** FDA and EMA approved indications of GLP-1 receptor agonists

Drug name	Pharmacology	FDA Approved Indications (US)	EMA Approved Indications (EU)
Exenatide	§ Exendin-4 analog § Subcutaneous BID or subcutaneous weekly	§ T2DM	§ T2DM
Liraglutide	§ Human GLP-1 analog § Long acting § Subcutaneous daily	§ T2DM § Reduction of MACE in T2DM patients with established cardiovascular disease § Chronic weight management	§ T2DM § Reduction of MACE in T2DM patients with established cardiovascular disease § Chronic weight management
Dulaglutide	§ Human GLP‑1 analog fused to IgG4 Fc fragment. § Subcutaneous Weekly	§ T2DM § Reduction of MACE in adults with T2DM and established cardiovascular disease or multiple risk factors	§ T2DM § Reduction of MACE in adults with T2DM and established cardiovascular disease or multiple risk factors
Semaglutide	§ Human GLP‑1 analog with fatty acid chain § Subcutaneous weekly § Oral daily	§ T2DM § Reduction of MACE in patients with established cardiovascular disease and either type 2 diabetes, obesity, or overweight § Chronic weight management § MASH in adults with moderate-to-advanced fibrosis § Kidney disease progression in T2DM patients with CKD	§ T2DM § T2DM patients with established cardiovascular disease § Chronic weight management
Lixisenatide	§ exendin-4 analog § Subcutaneous daily	§ T2DM	§ T2DM
Tirzepatide	§ Dual GLP-1/GIP agonist § Subcutaneous Weekly	§ T2DM § Chronic weight management § Obstructive sleep Apnea	§ T2DM § Chronic weight management

*FDA* United States Food and Drug Administration, *EMA* European Medicines Agency

*T2DM* Type 2 Diabetes Mellitus, *MASH* metabolic-associated steatohepatitis, *CKD* Chronic kidney disease, *MACE* Major adverse cardiovascular events

#### Are There Specific Patient Populations Who Might Benefit Most from GLP-Agonists?

The American Diabetes Association (ADA) and the European Association for the Study of Diabetes (EASD) recommend considering GLP-1R agonists for T2DM patients who do not have established CVD but are at high risk for ASCVD. High-risk indicators include age 55 years and above with significant narrowing—greater than 50%—in their coronary, carotid, or lower extremity arteries, left ventricular hypertrophy, estimated glomerular filtration rate (eGFR) < 60 mL/min/1.73 m², and albuminuria [121].

The ACC also supports the use of GLP-1R agonists for cardiovascular risk reduction in patients with T2DM and multiple ASCVD risk factors, independent of glucose control or background antihyperglycemic therapy [122]. The American Association of Clinical Endocrinologists (AACE) highlights that GLP-1R receptor agonists with proven cardiovascular benefits, such as liraglutide, semaglutide, and dulaglutide, should be considered for T2DM patients at high risk for ASCVD [123].

### Duration of Benefit

The health benefits of GLP-1R agonists are most pronounced within the first year of treatment, with significant improvements in glycated hemoglobin (HbA1c), body mass index (BMI), and cardiovascular outcomes. A retrospective study showed that HbA1c improved significantly during the first year of treatment and this effect was maintained over four years, although the benefits plateaued after the first year. The cardiovascular benefits, including reductions in MACE and stroke, also accumulate over time, with the most substantial gains observed within the first 24 to 36 months of treatment [124, 125].

### Barriers To GLP-1 Agonist Use

#### Understanding Benefits, Drug Costs and Broad Accessibility

While the data supporting a cardiovascular benefit for GLP-1 agonists are robust, their use in medical practice has been lower than expected worldwide. In 2017, a multicenter study of 313 sites showed that only 6% of patients who were eligible to receive GLP-1 agonists were given this class of medication [126]. Why is the prescribed use so low? There are several considerations including but not limited to high cost, a requirement for prior authorization, and denial of coverage [127]. Medication shortages have intermittently had an impact as well [128]. It is important to acknowledge inequitable access to GLP-1 agonists among patients of color and those with lower incomes [129].

We believe that the available data shows that a lack of knowledge about GLP-1R agonists benefits among physicians has immensely affected their use in clinical practice. A recent study conducted in an academic medical center determined the pattern of GLP-1 agonist use and showed that primary care physicians accounted for 45.1%, endocrinologists 45.0%, and cardiologists 1.4% of all prescriptions. In this analysis, prescription patterns were not affected by patient insurance status, suggesting that practitioner knowledge of benefit or indications for use were stark barriers [130]. Cardiologists often felt less comfortable prescribing “diabetes medications” [131].

Practitioners’ hesitancy to start new medications due to perceived titration complexity, incomplete understanding of drug interactions, and uncertainty of monitoring tools and goals of care, coupled with a belief that GLP-1R agonists requiring subcutaneous injections would not be well received by patients are major barriers to optimal use. Gastrointestinal side effects, the most commonly being nausea, might introduce further hesitancy. A recent study found that the overall 12-month discontinuation rate of GLP-1 agonists was 36.5%. The demographic distribution of those most likely to stop treatment was as follows: patients of color, men, people enrolled in Medicare or Medicaid, those living in areas with very high social needs, heart failure, obesity or other cardiovascular diseases prior to drug initiation, and those experiencing new gastrointestinal adverse effects at follow-up [132].

High out-of-pocket costs, complex cost-sharing structures, and gaps in prescription drug coverage are major healthcare policy barriers to medication use in underserved and older patient populations. Despite the closure of the Medicare Part D “donut hole,” many older adults still report financial hardship from prescription drug purchases, especially those with low income, multiple chronic conditions, or who take multiple medications daily. Even with insurance, cost-related nonadherence remains prevalent, with patients often skipping doses, delaying fills, or not obtaining needed medications due to cost [133, 134].

Policies that require significant coinsurance, deductibles, or lack of caps on annual out-of-pocket spending disproportionately affect these populations. For example, Medicare Part D plans still require beneficiaries to pay premiums and cost-sharing, and those without supplemental coverage or who do not qualify for the Low-Income Subsidy face higher financial barriers. The uptake of the Low-Income Subsidy remains suboptimal, and eligibility thresholds may exclude many at risk for nonadherence [135].

Recent policy interventions, such as the Inflation Reduction Act, aimed to expand eligibility for subsidies and cap out-of-pocket costs, but implementation and awareness challenges persist. Real-time benefit tools are now required in Medicare Part D to inform prescribers of patient-specific drug costs, but their effectiveness depends on clinician use and patient engagement in cost discussions [136].

Underserved populations, including those with food insecurity, disabilities, or poor health, are at even higher risk for cost-related nonadherence. Broader Medicaid eligibility, gradual tapering of cost-sharing assistance, and increased transparency in drug pricing are proposed policy solutions to reduce these barriers [137], but new legislation is expected to further depress the availability of beneficial drugs, including GLP-1R agonists, reversing attempts to optimize care for those in greatest need [138].

#### Bleeding Risk

The most common adverse reactions when combining GLP-1R agonists with traditional antithrombotic drugs such as aspirin, clopidogrel, or direct oral anticoagulants (DOACs) include gastrointestinal symptoms of nausea, vomiting, abdominal cramping, and increased bleeding risks. The risk of bleeding has not been a frequent topic of consideration or discussion amongst clinicians [139], but as previously summarized the GLP-1R agonists do have antithrombotic properties.

The combination of GLP-1 receptor agonists with aspirin or other antithrombotic agents can increase the risk of gastrointestinal bleeding. This is particularly concerning given that both drug classes can independently contribute to bleeding risks [140]. GLP-1 receptor agonists can delay gastric emptying, potentially affecting the absorption and pharmacokinetics of co-administered oral medications, including DOACs. This can lead to increased drug exposure and heightened risk of adverse effects, particularly with narrow therapeutic index drugs like dabigatran [141].

#### Patients at Risk for Bleeding

The elderly and patients with obesity are most affected by the increased bleeding risk when combining GLP-1R agonists with antithrombotic drugs such as aspirin, clopidogrel, or direct oral anticoagulants (DOACs). The risk of gastrointestinal bleeding increases significantly with age, especially in patients older than 75 years. Combination antithrombotic therapy, including GLP-1 receptor agonists, further exacerbates this risk [142].

Individuals with obesity, both with and without T2DM, show increased risks for gastrointestinal bleeding when treated with GLP-1R agonists combined with aspirin [140]. The mechanism(s) has not been delineated, and further investigation is needed.

Patients with multiple comorbidities, such as atrial fibrillation or venous thromboembolism, are at higher risk of bleeding when using DOACs in combination with GLP-1R agonists. Drug-drug interactions and the presence of other medications further increase the bleeding risk [143].

### Addressing Current Gaps and Unmet Needs

GLP-1R agonists have rapidly evolved from glucose-lowering agents for T2DM to multifaceted therapeutics with FDA- and EMA-approved indications for obesity, cardiovascular disease, chronic kidney disease, diabetic nephropathy, and sleep apnea. However, significant gaps remain in translating these benefits into optimal patient care and population health [144]. These include limited long-term safety data across diverse populations, insufficient understanding of mechanisms in neuropsychiatric and oncologic contexts, and suboptimal communication between clinicians and patients. Moreover, disparities in access and coverage, particularly for oral formulations and newer agents, hinder equitable implementation. Targeted research addressing these gaps, especially in real-world effectiveness, risk stratification, and health equity, will be essential to fully realize the population-level potential of GLP-1R agonists.

Grounded in current scientific understanding, established mechanisms of action, and emerging clinical data, we recognize a broad spectrum of medical conditions and critical domains that warrant further exploration and targeted investigation to fully realize the therapeutic potential of GLP-1R agonists.

1. *Cardiovascular-Kidney-Metabolic (CKM) Syndrome*, as defined by the AHA, is a systemic disorder characterized by pathophysiological interactions among metabolic risk factors (such as obesity, insulin resistance, dyslipidemia, and hypertension), chronic kidney disease (CKD), and the cardiovascular system, leading to multiorgan dysfunction and a high rate of adverse cardiovascular outcomes [145]. The syndrome encompasses both individuals at risk for ASCVD due to the presence of metabolic risk factors or CKD, and those with established CVD complicated by metabolic or kidney dysfunction [146]. Recent U.S. data indicate that approximately 90% of adults meet criteria for a CKM stage, with higher prevalence in older adults and in men, and a projected further increase due to the ongoing epidemics of obesity and diabetes [147].
2. *Primary cardiovascular disease prevention* is an important area of investigation. Although a prior cohort study did not show a statistically significant reduction in major cardiovascular events among patients with no previous history of cardiovascular disease [148], a meta-analysis of eight clinical trials evaluating the effect of GLP-1R agonists on cardiovascular outcomes reported a 14% reduction in major adverse cardiovascular events, with no significant heterogeneity across GLP-1R agonists or subgroups, including patients with or without previous cardiovascular disease [149]. The ACC recommends GLP-1R agonists for cardiovascular risk reduction in patients with T2DM and established ASCVD [114]. This benefit is also supported by trials such as LEADER, SUSTAIN-6, and EXSCEL, in which the reduction in MACE was significant among those with established ASCVD. The REWIND trial with dulaglutide included many patients without established ASCVD and demonstrated a consistent reduction in MACE, supporting a role in primary prevention, although the effect size was modest. The SOUL and FLOW trials further support these findings for oral semaglutide. Importantly, the cardiovascular benefit appears to be independent of glycemic control and is observed across subgroups defined by age, sex, and BMI [113, 150]. However, not all agents in the class are equivalent; short-acting agents such as lixisenatide and exenatide have not demonstrated cardiovascular benefit [151]. The variability in benefit within and across agents in the GLP-1R agonist class remains an important area of investigation.
3. *Metabolic dysfunction–associated steatohepatitis (MASH*). GLP-1R agonists are known to have beneficial effects in this large and steadily growing patient population, primarily through their effects on weight loss and insulin secretion, although emerging evidence suggests direct hepatic effects as well [152, 153]. Because MASH is a significant risk factor for cardiovascular disease and events, this remains an important area for future investigation [154].
4. *Microvascular disease and microvascular dysfunction*. The known microvascular diseases and disorders encompass a broad spectrum of conditions affecting small blood vessels (arterioles, capillaries, and venules) across multiple organ systems. The most well-established microvascular diseases and disorders include diabetic microvascular complications, small cerebral vessel disease, renal microvascular disease, pulmonary microvascular disease, systemic autoimmune microvascular disorders, and hereditary microangiopathies [155–158].

GLP-1RAs attenuate pathological processes such as oxidative stress and inflammation and improve endothelial function—both central to microvascular injury in diabetes and related conditions. These agents enhance nitric oxide (NO) production and endothelial nitric oxide synthase (eNOS) phosphorylation, leading to vasodilation and increased microvascular perfusion in both diabetic and non-diabetic individuals [159, 160].

GLP-1R agonists increase microvascular recruitment and blood flow in skeletal and cardiac muscle, even in the presence of insulin resistance, which may improve tissue oxygenation and metabolic responses [161]. In animal models, GLP-1R agonists prevent capillary rarefaction and microvascular barrier dysfunction and reduce apoptosis and reactive oxygen species in microvascular endothelial cells, contributing to improved organ function and reduced progression of microvascular complications [162].

Clinical trials have shown that GLP-1R agonists increase microvascular blood volume and flow in cardiac muscle, and these effects are preserved in obese individuals with vascular insulin resistance. In addition, systematic reviews and meta-analyses of randomized controlled trials report improvements in cardiac systolic and diastolic function in patients with type 2 diabetes, as well as reductions in infarct size after myocardial infarction [163].

Direct clinical trial evidence that GLP-1R agonists reduce symptoms and clinical events specifically in patients with coronary microvascular dysfunction is limited. Most cardiovascular outcome trials and meta-analyses have focused on broader populations with type 2 diabetes and established cardiovascular disease, rather than on patients with isolated coronary microvascular dysfunction [164]. Given the prevalence of coronary microvascular dysfunction, further investigation into the effects of GLP-1R agonists is warranted.

5. *Combination Therapies*: in addition to dual or triple GLP-1R agonist therapy and GLP-1R agonist and gastrointestinal peptide (GIP) therapy [165, 166], nterest has emerged in the combined use of cagrilintide—a long-acting amylin analogue that, as monotherapy, produces clinically meaningful weight loss and is well tolerated—with a GLP-1R agonist. Coadministration of cagrilintide and semaglutide (CagriSema) at 2.4 mg each, once weekly, provides substantial benefits for patients with overweight, obesity, or T2DM. The combination yields greater weight loss than either agent alone, with mean reductions in body weight of approximately 20.4% over 68 weeks in non-diabetic adults and 13.7% in those with type 2 diabetes—far exceeding placebo and monotherapy arms. Cardiovascular benefits include significant reductions in systolic and diastolic blood pressure (mean systolic reduction of − 9.9 mm Hg in non-diabetic adults and − 6.5 mm Hg in those with diabetes), improved lipid profiles, and lower C-reactive protein levels, all of which are relevant for cardiovascular risk reduction. Additionally, glycemic control is markedly improved, with a higher proportion of patients achieving normoglycemia or HbA1c ≤ 6.5% [167, 168].
6. *Maintaining muscle mass while losing weight*. GLP-1R agonists consistently cause a reduction in muscle mass (usually measured as lean mass or fat-free mass) as part of their overall weight-loss effect. The magnitude of muscle mass loss varies by agent, population, and study methodology, but typically comprises 15–40% of total weight lost, with most studies reporting 15–30%. The time course of muscle mass loss generally parallels the rate of weight loss, with the most rapid changes occurring in the first 3–4 months of treatment, particularly with higher-dose agents such as semaglutide and tirzepatide. There is variability across drugs [169–172].

Loss of muscle mass can be minimized or prevented through several key strategies, including structured resistance or strength training, ensuring adequate dietary protein intake, and incorporating regular exercise. A slower, more moderate pace of weight loss, along with careful attention to nutritional status, is particularly important for older adults or individuals at risk for sarcopenia [173].

Adjunctive therapies such as bimagrumab and myostatin inhibitors are currently under investigation. These agents block the activin type II receptors (ActRIIA and ActRIIB), which are key mediators of muscle catabolism through the myostatin and activin A signaling pathways. GLP-1R agonists increase signaling through ActRIIA/B via myostatin and activin A [174]. Bimagrumab is a monoclonal antibody that binds to both ActRIIA and ActRIIB, preventing myostatin and activin A from activating these receptors. This blockade inhibits the downstream Smad2/3 pathway—which otherwise promotes muscle protein degradation and atrophy—and instead promotes muscle hypertrophy and differentiation [175]. Dual blockade of both ActRIIA and ActRIIB is necessary for maximal anabolic response and muscle preservation, as single-receptor inhibition is insufficient. In preclinical models, co-administration of bimagrumab or dual myostatin/activin A inhibitors with GLP-1R agonists preserves or increases muscle mass while enhancing fat loss, resulting in improved body composition and metabolic outcomes compared with GLP-1R agonist monotherapy [176]. This effect is mediated by both Akt-dependent and Akt-independent pathways, supporting muscle growth even during caloric deficit [177].

#### Ongoing Research and Development

GLP-1R agonists have demonstrated significant cardiovascular benefits in addition to effectively lowering blood glucose levels, including major cardiovascular events risk reduction. Ongoing research and development strategies focus on enhancing efficacy and convenience, such as developing dual and triple receptor agonists and new formulations for oral administration or extended dosing schedules. In addition, studies are investigating their broader benefits in patients with chronic kidney disease (CKD) and in elderly populations, with the aim of improving overall health outcomes in these high-risk groups Table 3.

**Table 3:** Ongoing and recently completed clinical trials of GLP-1 agonists

Trial Name	Abbreviated Name	GLP-1 Agonist & Dose	Design	Endpoints	Anticipated Completion Date
Danuglipron Phase 2b Study in Obesity	**NCT04707313**	Danuglipron (40 mg to 200 mg twice daily)	Randomized, double-blind, placebo-controlled	Primary: Change in body weight; Secondary: Safety and tolerability, HbA1c levels	*End of 2024*
Danuglipron Phase 2 Study in Type 2 Diabetes	**NCT03985293**	Danuglipron (2.5 mg to 120 mg twice daily)	Randomized, double-blind, placebo-controlled	Primary: Change in HbA1c; Secondary: Fasting plasma glucose, body weight, safety	*Completed*
Once-Daily Modified Release Danuglipron Study	**NCT06153758**	Danuglipron (dose optimization ongoing)	Open-label, randomized	Primary: Pharmacokinetics; Secondary: Safety, dose optimization	*Second half of 2024*
SURPASS CVOT	**NCT04255433**	Tirzepatide (5 mg, 10 mg, 15 mg weekly)	Randomized, double-blind, placebo-controlled	Primary: Composite of MI, stroke, and CV death; Secondary: All-cause mortality, hospitalization for heart failure	June 2025
SURMOUNT MMO	**NCT05556512**	Tirzepatide (5 mg, 10 mg, 15 mg weekly)	Randomized, double-blind, placebo-controlled	Primary: Composite of all‑cause mortality and CV events. Secondary: % weight change, waist circumference, HbA1c, lipids, safety.	2027
Soul	**NCT06659718**	Oral semaglutide vs. sitagliptin	Observational, retrospective.	Primary: Time to first occurrence of MACE. Secondary: Expanded MACE, all‑cause mortality, HF hospitalization, renal outcomes	Completed
Retatrutide – TRIUMPH‑Outcomes	**NCT06383390**	Retatrutide SC weekly	randomized, double‑blind, placebo‑controlled	Primary: Composite of MACE or major adverse kidney event. Secondary: All‑cause mortality, HF hospitalization, renal function, weight change	2029
Orforglipron – ATTAIN‑2	**NCT06824051**	Orforglipron 6 mg, 12 mg, 36 mg oral daily	randomized, double‑blind, placebo‑controlled	Primary: % weight change at Week 72. Secondary: HbA1c change, % achieving HbA1c ≤ 6.5%, CV risk factors, hsCRP, safety	2027
HRS9531	**NCT06841445**	HRS9531 oral tablet	randomized, double‑blind, placebo‑controlled	Primary: % weight change at Week 26. Secondary: Waist circumference, metabolic markers, safety	Dec 2025
CT‑868	**NCT06062069**	CT‑868 SC daily (≤ 6.6 mg)	randomized, quadruple‑blind, placebo‑controlled	Primary: HbA1c change at Week 16. Secondary: Weight, insulin dose, CGM metrics, safety	2025

There are several ongoing studies. The SOUL trial (NCT06659718) will investigate the effect of semaglutide on cardiovascular outcomes in patients with T2DM who have preexisting cardiovascular disease and/or CKD. The SURPASS trial (NCT04093752) will compare tirzepatide with liraglutide in high-risk patients with T2DM, while the SURMOUNT MMO study (NCT05556512) will evaluate the impact of tirzepatide on morbidity and mortality in patients with obesity.

Novel GLP-1R agonists currently under development include HRS9531, a dual GLP-1/GIP receptor agonist being evaluated in overweight or obese individuals with T2DM, which has shown promising results in a Phase 2 study [172]. CT-388 and CT-868, developed by Carmot Therapeutics, are GIPR/GLP-1R agonists under investigation in obese patients, including those with type 1 diabetes (NCT04838405, NCT06062069).

Retatrutide is a triple agonist of the GIP, GLP-1, and glucagon receptors that is currently in development. Investigations to date have demonstrated promising safety and efficacy profiles [178]. Danuglipron and orforglipron are nonpeptide oral GLP-1R agonists in development that have shown encouraging safety and efficacy in glycemic control among patients with T2DM [179].

### Defining a Path Forward for Clinical Advances

To fully realize the transformative potential of GLP-1R agonists in both individualized care and public health, a concerted effort is needed from clinicians, researchers, regulators, and industry stakeholders [144]. Clinicians are uniquely positioned to identify patient-specific needs and integrate GLP-1R agonists into personalized treatment plans, especially for those with T2DM, obesity, and related metabolic conditions. Their role in educating patients, monitoring outcomes, and advocating for equitable access is critical. Researchers must continue to explore the multifaceted effects of GLP-1R agonists across organ systems, investigate long-term safety and efficacy, and uncover genetic and environmental factors that influence therapeutic response. Regulators can accelerate access by embracing regulatory reliance and convergence, streamlining approval processes, and fostering trust through transparent and collaborative frameworks. Industry stakeholders must prioritize affordability and sustainability, expanding production and distribution models that serve underserved populations. This includes investing in generic manufacturing, supporting localized supply chains, and aligning innovation with equity goals. They should listen carefully to both the clinical and scientific communities and support investigations in new and emerging areas. Together, these groups can close existing gaps in accessibility and ensure that GLP-1R agonists are not only available but also effectively and optimally utilized to improve health outcomes across diverse communities.

## Conclusions

The expanding role of GLP-1R agonists in contemporary clinical practice underscores their multifaceted benefits across cardiovascular and metabolic diseases. These agents have demonstrated significant efficacy in reducing cardiovascular events, improving metabolic parameters, and offering protective effects against inflammation and atherosclerosis. They also possess antithrombotic properties that can be leveraged with complementary medications used in patients with coronary artery disease, including those with prior events. Despite robust clinical evidence, barriers such as high costs, limited access, lack of awareness of approved indications, and practitioner hesitancy continue to impede widespread adoption.

Practitioners are encouraged to follow existing guidelines and engage in continuing education to better understand the evolving therapeutic landscape. Ongoing research and development efforts, including novel formulations, dual- and triple-agonist therapies, and extended-release oral agents, hold promise for enhancing accessibility, tolerability, and sustained benefits.

Furthermore, emerging data support expanded indications in non-diabetic populations, patients with CKM syndrome, microvascular dysfunction, and metabolic liver disease. Strategies to preserve muscle mass and optimize body composition during treatment are also under active investigation. To fully realize the translational potential of GLP-1R agonists, future efforts must address gaps in implementation and integrate real-world evidence into clinical decision-making. As our mechanistic understanding deepens and therapeutic applications broaden, GLP-1R agonists are poised to become cornerstone agents in the prevention and management of cardiometabolic diseases, ultimately improving patient outcomes and public health.

## Key References

- Pandey S, Mangmool S, Parichatikanond W. Multifaceted Roles of GLP-1 and Its Analogs: A Review on Molecular Mechanisms with a Cardiotherapeutic Perspective. Pharmaceuticals. 2023;16(6):836.
  - Review of GLP-1 and its analogs mechanisms in the protection against cardiomyopathies.
- Rivera, K., et al., The Gut-Heart Axis: Molecular Perspectives and Implications for Myocardial Infarction. Int J Mol Sci, 2024. 25(22).
  - Evidence of gastrointestinal- cardiovascular system signaling.
- Ben Nasr M, Usuelli V, Dellepiane S, Seelam AJ, Fiorentino TV, D'Addio F, et al. Glucagon-like peptide 1 receptor is a T cell-negative costimulatory molecule. Cell metabolism. 2024;36(6):1302-19.e12.
  - GLP-1 receptor role as a negative costimulatory mechanism, mitiagting alloimmune responses.
- Ussher JR, Drucker DJ. Glucagon-like peptide 1 receptor agonists: cardiovascular benefits and mechanisms of action. Nature reviews Cardiology. 2023;20(7):463-74.
  - Immune modulation effect of GLP-1 agonist.
- Bendotti G, Montefusco L, Lunati ME, Usuelli V, Pastore I, Lazzaroni E, et al. The anti-inflammatory and immunological properties of GLP-1 Receptor Agonists. Pharmacological research. 2022;182:106320.
  - Anti-Inflammatory benefit of GLP-1 agonist.
- Lee J, Hong S-W, Kim M-J, Moon SJ, Kwon H, Park SE, et al. Glucagon-like peptide receptor agonist inhibits angiotensin II-induced proliferation and migration in vascular smooth muscle cells and ameliorates phosphate-induced vascular smooth muscle cells calcification. Diabetes & Metabolism Journal. 2024;48(1):83-96.
  - Effects of GLP-1 agonist on vascular smooth muscle cells.
- Zhou, Z.D., et al., Glucagon-like peptide-1 receptor agonists in neurodegenerative diseases: Promises and challenges. Pharmacol Res, 2025. 216: p. 107770.
  - Neuroprotective effect of GLP-1 agonists.
- Attia SM, Alshamrani AA, Ahmad SF, Albekairi NA, Nadeem A, Attia MS, et al. Dulaglutide reduces oxidative DNA damage and hypermethylation in the somatic cells of mice fed a high‐energy diet by restoring redox balance, inflammatory responses, and DNA repair gene expressions. Journal of Biochemical and Molecular Toxicology. 2024;38(7):e23764.
  - The effect of GLP-1 agonist on vasculature and vascular aging.
- Greco C, Santi D, Brigante G, Pacchioni C, Simoni M. Effect of the glucagon-like peptide-1 receptor agonists on autonomic function in subjects with diabetes: a systematic review and meta-analysis. Diabetes & Metabolism Journal. 2022;46(6):901-11.
  - Multiorgan effect of GLP-1 agonists contributing to cardiovascualr benifits.
- Liu G, Liao W, Lv X, Huang L, He M, Li L. A potential coagulation-related diagnostic model associated with immune infiltration for acute myocardial infarction. Genes & Immunity. 2024:1-12
  - Effect of coagulation and immune responses in acute myocardail infarction could lead to the development of more targeted therapies.
- Yen F-S, Hou M-C, Wei JC-C, Shih Y-H, Hwu C-M, Hsu C-C. Effects of glucagon-like peptide-1 receptor agonists on liver-related and cardiovascular mortality in patients with type 2 diabetes. BMC medicine. 2024;22(1):8
  - Effect of GLP-1 agonist on improving cardiovascular function through improving liver function.
- Piccini S, Favacchio G, Panico C, Morenghi E, Folli F, Mazziotti G, et al. Time-dependent effect of GLP-1 receptor agonists on cardiovascular benefits: a real-world study. Cardiovascular diabetology. 2023;22(1):69.
  - Highlight cardioprotective mechanisms of action of GLP-1 agonist.
- Ferhatbegović L, Mršić D, Macić-Džanković A. The benefits of GLP1 receptors in cardiovascular diseases. Frontiers in Clinical Diabetes and Healthcare. 2023;4.
  - Highlight cardioprotective mechanisms of action of GLP-1 agonist.
- Drucker DJ. The GLP-1 journey: from discovery science to therapeutic impact. The Journal of clinical investigation. 2024;134(2).
  - GLP-1 agonists effect on augmenting fibrinolytic potential.
- Mashayekhi M, Beckman JA, Nian H, Garner EM, Mayfield D, Devin JK, et al. Comparative effects of weight loss and incretin-based therapies on vascular endothelial function, fibrinolysis and inflammation in individuals with obesity and prediabetes: A randomized controlled trial. Diabetes, Obesity and Metabolism. 2023;25(2):570-80.
  - Evidence of anti thrombotic effect of GLP-1 agonsit.
- Deng G, Ren J, Li R, Li M, Jin X, Li J, et al. Systematic investigation of the underlying mechanisms of GLP-1 receptor agonists to prevent myocardial infarction in patients with type 2 diabetes mellitus using network pharmacology. Frontiers in pharmacology. 2023;14.
  - Review of the potential mechanisms underlying the cardiovascular benefits of GLP-1RAs in T2DM patients.
- Gerstein HC, Li Z, Ramasundarahettige C, Baek S, Branch KRH, Del Prato S, et al. Exploring the Relationship Between Efpeglenatide Dose and Cardiovascular Outcomes in Type 2 Diabetes: Insights From the AMPLITUDE-O Trial. Circulation. 2023;147(13):1004-13.
  - Dose dependent response effect of GLP-1 agonist.
- Lincoff AM, Brown-Frandsen K, Colhoun HM, Deanfield J, Emerson SS, Esbjerg S, et al. Semaglutide and Cardiovascular Outcomes in Obesity without Diabetes. The New England journal of medicine. 2023;389(24):2221-32.
  - The first study to show efficacy of GLP-1 agonist in patients with preexisting cardiovascular disease and overweight or obese without diabetes.
- Caruso P, Maiorino MI, Longo M, Porcellini C, Matrone R, Digitale Selvaggio L, et al. Liraglutide for Lower Limb Perfusion in People With Type 2 Diabetes and Peripheral Artery Disease: The STARDUST Randomized Clinical Trial. JAMA Netw Open. 2024;7(3):e241545.
  - Potinital GLP-1 use to pevent the clinical progression of PAD in individuals with type 2 diabetes.
- 9.Pharmacologic Approaches to Glycemic Treatment: Standards of Medical Care in Diabetes-2022. Diabetes care. 2022;45(Suppl 1):S125-s43.
  - Latest ADA recommendations for GLP-1 agonist use.
- Morton JI, Marquina C, Shaw JE, Liew D, Polkinghorne KR, Ademi Z, et al. Projecting the incidence and costs of major cardiovascular and kidney complications of type 2 diabetes with widespread SGLT2i and GLP-1 RA use: a cost-effectiveness analysis. Diabetologia. 2023;66(4):642-56.
  - Cost-effectivness of GLP-1 agonist use.
- Tanne JH. Wegovy: FDA approves weight loss drug to cut cardiovascular risk. Bmj. 2024;384:q642.
  - FDA approval for sumaglutide for cardiovascular benefit in patient without Diabeteis.
- FDA Approves First Medication for Obstructive Sleep Apnea. 2024 [cited 2025 December, 20]; Available from: https://www.fda.gov/news-events/press-announcements/fda-approves-first-medication-obstructive-sleep-apnea.
  - FDA approval for GLP-1 agonist in obstructive sleep apnea.
- FDA Approves Treatment for Serious Liver Disease Known as ‘MASH’. 2025 [cited 2-25 September 1]; Available from: https://www.fda.gov/drugs/news-events-human-drugs/fda-approves-treatment-serious-liver-disease-known-mash.
  - FDA approval for GLP-1 Agonist in patients with MASH.
- Blonde L, Umpierrez GE, Reddy SS, McGill JB, Berga SL, Bush M, et al. American Association of Clinical Endocrinology Clinical Practice Guideline: Developing a Diabetes Mellitus Comprehensive Care Plan-2022 Update. Endocr Pract. 2022;28(10):923-1049.
  - Latest AACE guidelines for GLP-1 agonists use.
- King A, Miller EM. Glucagon-Like Peptide 1 Receptor Agonists Have the Potential to Revolutionize the Attainment of Target A1C Levels in Type 2 Diabetes-So Why Is Their Uptake So Low? Clin Diabetes. 2023;41(2):226-38.
  - Barriers to GLP-1 agonist use.
- Do D, Lee T, Peasah SK, Good CB, Inneh A, Patel U. GLP-1 Receptor Agonist Discontinuation Among Patients With Obesity and/or Type 2 Diabetes. JAMA Netw Open. 2024;7(5):e2413172.
  - Review of causes of medication discontiuation.
- Makam, A.N., et al., Availability of Cardioprotective Medications for Type 2 Diabetes in the Medicaid Program. Annals of Internal Medicine, 2025. 178(6): p. 808-818.
  - Barriers to availability of GLP-1 agonists.
- Lin, C.M., et al., Unexpected cardiovascular risks of glucagon-like peptide-1 receptor agonist and aspirin co-administration in individuals with obesity, with and without type 2 diabetes: A propensity score matched cohort study. Diabetes Obes Metab, 2025. 27(4): p. 1980-1991.
  - Bleeding risk in glucagon-like peptide-1 receptor agonist and aspirin co-administration.
- Hooper L, Liu S, Pai MP. GLP-1RA-induced delays in gastrointestinal motility: Predicted effects on coadministered drug absorption by PBPK analysis. Pharmacotherapy. 2025.
  - Highlight the effect of GLP-1 agonist on pharmacokinetics of oral medications by delaying gastric emptying.
- Ndumele, C.E., et al., A Synopsis of the Evidence for the Science and Clinical Management of Cardiovascular-Kidney-Metabolic (CKM) Syndrome: A Scientific Statement From the American Heart Association. Circulation, 2023. 148(20): p. 1636-1664.
  - Defenition of CKM and relation to cardiovascular outcomes.
- Tang, A.S.P., et al., Glucagon-like peptide-1 receptor agonist in myocardial infarction and atherosclerotic cardiovascular disease risk reduction: a comprehensive meta-analysis of number needed to treat, efficacy and safety. Cardiovasc Diabetol, 2025. 24(1): p. 285.
  - Evidence of GLP-1 agonist cardiovascular benefit acroos all subgroups independent of glycemic control.
- Zhang, J., et al., Novel Dual and Triple Agonists Targeting GLP-1, GIP, Glucagon, and GDF15 for Type 2 Diabetes and Obesity Management. Endocrinology, 2025.
  - Highlights Dual and triple Agonists therapy.
- Garvey, W.T., et al., Coadministered Cagrilintide and Semaglutide in Adults with Overweight or Obesity. New England Journal of Medicine, 2025. 393(7): p. 635-647.
  - Emergance of combination therapy.
- Davies, M.J., et al., Cagrilintide–Semaglutide in Adults with Overweight or Obesity and Type 2 Diabetes. New England Journal of Medicine, 2025. 393(7): p. 648-659.
  - Efficacy of combination therapy in Obesity and T2DM.
- Anyiam, O., et al., How do glucagon-like Peptide-1 receptor agonists affect measures of muscle mass in individuals with, and without, type 2 diabetes: A systematic review and meta-analysis. Obes Rev, 2025. 26(7): p. e13916.
  - Effect of GLP-1 agonist on muscle mass.
- Mastaitis, J.W., et al., GDF8 and activin A blockade protects against GLP-1-induced muscle loss while enhancing fat loss in obese male mice and non-human primates. Nat Commun, 2025. 16(1): p. 4377.
  - Pathway blockade to reduce muscle mass loss in GLP-1 agonists use.
- Nunn, E., et al., Antibody blockade of activin type II receptors preserves skeletal muscle mass and enhances fat loss during GLP-1 receptor agonism. Mol Metab, 2024. 80: p. 101880.
  - Evidence of Monoclonal antibody to prsereve muscle mass in GLP-1 use.
- Jastreboff, A.M., et al., Triple-Hormone-Receptor Agonist Retatrutide for Obesity - A Phase 2 Trial. N Engl J Med, 2023. 389(6): p. 514-526.
  - Efficacy of Triple Agonist under development.
- Frias JP, Hsia S, Eyde S, Liu R, Ma X, Konig M, et al. Efficacy and safety of oral orforglipron in patients with type 2 diabetes: a multicentre, randomised, dose-response, phase 2 study. Lancet. 2023;402(10400):472-83.
  - Latest study showing efficacy of nonpeptide oral GLP-1R agonists currently under development.

## Data Availability

No datasets were generated or analysed during the current study.
